# *MoMa*: An assistive mobile manipulator with a webcam-based gaze control system

**DOI:** 10.1016/j.ohx.2024.e00599

**Published:** 2024-10-19

**Authors:** James Dominic O. Go, Neal Garnett T. Ong, Carlo A. Rafanan, Brian G. Tan, Timothy Scott C. Chu

**Affiliations:** De La Salle University Manila, 2401 Taft Ave, Malate, Manila, 1004 Metro Manila, Philippines

**Keywords:** Convolutional neural networks, Gaze control, Mobile manipulator, Unmanned ground vehicle, Webcam

## Abstract

*Mobile Manipulators (MoMa) is a category of mobile robots* designed to assist people with motor disabilities to perform object retrieval tasks using a webcam-based gaze control system. Using off-the-shelf components such as reproducible acrylic and 3D-printed plates, and a webcam for eye tracking, *MoMa* serves as an inexpensive, open-source, and customizable solution in assistive robotics. The robotic system consists of a mobile base that can move forward and backward, as well as turn in place; and a 2-axis cartesian arm equipped with a claw gripper that opens and closes. The simple movement of the robot also allows for a simple control method and graphical user interface (GUI). The user receives information about what is in front of the robot through a mounted camera, and, by looking at parts of the screen that correspond to controls, has their gaze predicted by a convolutional neural network and sends commands wirelessly. The performance of the entire system has been validated through testing of the gaze prediction model, the integration of the control system, as well as its task completion capabilities. All the design, construction and software files are freely available online under the CC BY 4.0 license at https://doi.org/10.17632/k7yfn6wdv7.2.

## Graphical abstract

**Figure:**
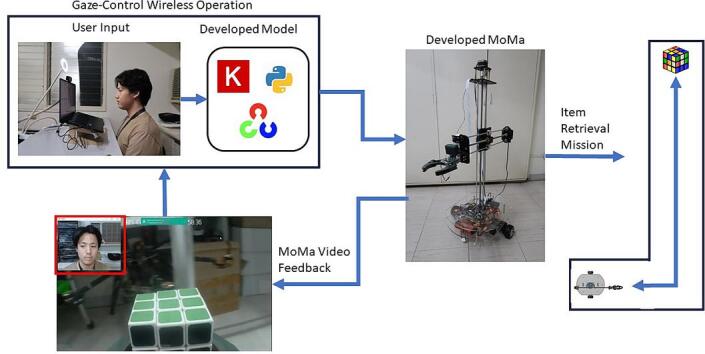

**Specifications Table:** 

**Hardware name**	*MoMa: An Assistive Mobile Manipulator with a Webcam-based Gaze Control System*
**Subject area**	● Engineering and materials science Robotics engineering Machine Learning and Computer Vision
**Hardware type**	● Assistive Mobile Manipulator Gaze Control System
**Closest commercial analog**	Stretch ($17950), [6], Gazepoint GP3 ($845) [22]
**Open source license**	Creative Commons BY 4.0.
**Cost of hardware**	Php 35159.15 ($632)
**Source file repository**	https://doi.org/10.17632/k7yfn6wdv7.2

## 1 Hardware in context

Throughout the years, development in the field of robotics has led to the creation of more efficient and effective robotic systems that can accomplish complex tasks. One application of robotic systems that has rapidly grown over the years is its implementation as an assistive system for people with disabilities. These assistive robotic systems are capable of assisting in tasks such as scratching, shaving, object retrieval, and telepresence [1].

**Fig. 1:**
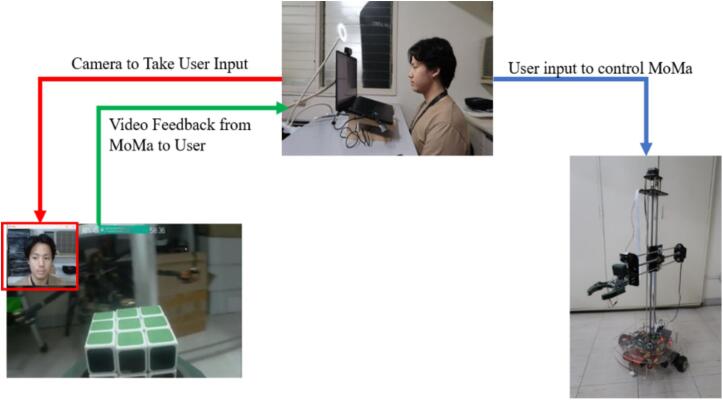
Set up for the MoMa mobile manipulator with gaze control system.

The general design of control systems utilized in operating robotic systems often require inputs such as key or button presses, mouse movements, and joystick movements. These required inputs generally require the operator or user to have proper motor coordination to issue commands properly and accurately through the control system. This requirement creates a limitation for people with motor disabilities as they lack the capability and capacity to perform the necessary actions required in utilizing the control systems with their hands. A proposed method of control that is intuitive and can overcome the said limitation is the gaze control [2]. However, gaze control systems utilize eye trackers to detect the location of gaze, which is expensive and inaccessible for most people. A viable and inexpensive alternative to using eye trackers is to instead utilize a webcam-based gaze control system [3], [4]. With these considerations, we propose the MoMa, an assistive mobile manipulator with a webcam-based gaze control system. Fig. 1. Illustrates the setup of the MoMa system.

MoMa is similar to other existing robots like JACO [5] and the Stretch [6], but integrates a web-cam based eye tracking system to provide people with motor disabilities a method of control while addressing the issue of eye tracker cost. Additionally, the proposed manipulator system is designed to be modular with off-the-shelf components which help drive down the system’s total cost.

## 2 Hardware description

MoMa is an assistive mobile manipulator that is operated using a webcam-based gaze control system. MoMa is built using off-the-shelf components, and some easily reproducible fabrications, and may be reproduced at a low cost. This section details the robot’s hardware specifications and the software behind the control system.

### 2.1 Hardware description

The body of the mobile manipulator consists of mechanical and electronic components that are commercially available, and structures that are reproducible using 3D printing and acrylic laser cutting. The robotic system is designed to assist users with motor disabilities through its ability to complete object retrieval tasks. As such, the robot consists of a mobile base that can traverse indoor environments, and an arm equipped with a gripper that can reach and grasp common household items. A summary of the technical specifications of the robot is shown in Table 1.

**Table 1:** Mobile manipulator specifications.

Parameter	Specification
Total weight	Arm (3.2 kg) + Mobile Base (4.4 kg) = 7.6 kg
Dimensions (W × L × H)	35 cm × 65 cm × 100 cm
Maximum vertical reach	75 cm
Minimum vertical reach	20 cm
Maximum horizontal reach	20 cm (past mobile base)
Design speed	15 cm/s
Maximum speed	73 cm/s
Sensors	MY-37 hall encoders
Design payload	1 kg
Degrees of freedom of Manipulator	3 (2 translational axes + 1 gripper opening and closing)
Battery life	6 h (using 5600 mAh battery)

The mobile base is equipped with two fixed differential drive wheels driven by 12 V gear motors with hall encoders, and two ball-type omnidirectional wheels used for stability and maneuverability. Acrylic was chosen for its structure because it is stiff and strong to support the weight of the robot and may easily and precisely be shaped and cut according to specified design requirements. A clear material used to house the associated electronics is also helpful for accessibility and management. Allowing for a larger and wider mobile base platform, the positioning of the wheels may be more spread out to support the robot and prevent it from tipping, and the electronics may be laid out to be more spacious and for proper weight distribution. Additional space also accommodates additional parts that users may require.

The arm of the robot is a 2-axis cartesian type arm, equipped with a claw gripper. This simple design enables the robotic arm to move with only 3 degrees of freedom, allowing for a simpler gaze control system which will be discussed in the next section. The arm consists of a vertical and a horizontal linkage, which are both driven by lead screws. Lead screws were used in favor of a belt-driven actuation as it would allow the design to directly place the motors in-line with the lead screw for a more compact design. Two NEMA 17 stepper motors drive the lead screws, which are rotated to cause linear translation of the linkages. Its structure uses plates that are 3D-printed using PLA, which can withstand applied loads and be produced using additive manufacturing which enables precise customized fabrication. Customized plates may also be created to modify the system.

MoMa is equipped with a Raspberry Pi 4 (RPi 4) as its controller. The RPi 4 comes with numerous GPIO pins that can be used to control the wheel and stepper motors. It also has wireless networking capabilities that enable the robot to be controlled remotely. The software and control system is discussed in the following section.

### 2.2 Control system and software description

**Fig. 2:**
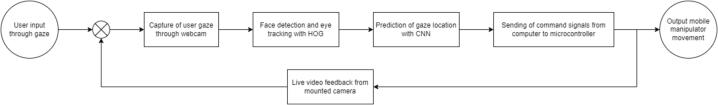
Control system framework.

**Fig. 3:**
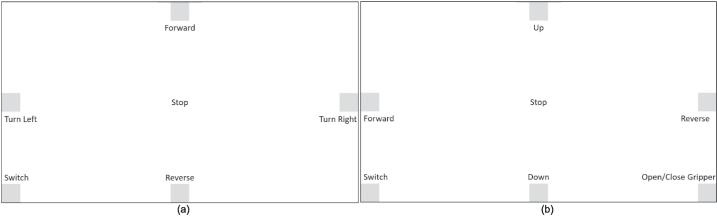
Control system GUI for (a) mobile base and (b) manipulator.

The gaze control system for the mobile manipulator consists of the webcam, the host computer, and the Raspberry Pi 4 (RPi4). User input comes in the form of gazing at specific portions on the screen. The webcam captures images of the user and sends it to the host computer, which then utilizes machine learning models to isolate images of the right eye and predict the location of gaze. Each specific location/portion of the screen is tied to a command for the robot. Users must maintain their gaze on a zone for a dwell time of 1 s before a command is activated. Dwell time is introduced to prevent any accidental triggering of commands. When gaze location is determined, the host computer sends a signal over WiFi to the RPi4, which will then actuate the mobile manipulator using its GPIO pins. The RPi4 also provides visual feedback via a camera mounted on the robot. Live feed is sent to the host computer over WiFi so that users are able to see the environment directly in front of the robot. The control system framework is summarized in Fig. 2. Fig. 3 also shows the control system GUI for (a) the mobile base and (b) the manipulator. The command tied to each zone is specified, where gazing at one zone triggers the command. Control can be switched between the mobile base and the manipulator through the lower left zone.

**Fig. 4:**
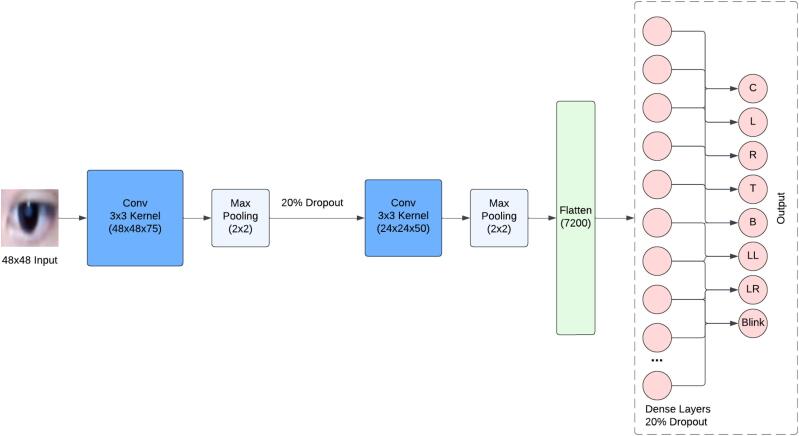
System architecture of the convolutional neural network.

The control system utilizes Python and Python libraries for all of its functions. OpenCV is used to capture and manipulate images from the webcam. For face and eye detection, dlib’s Histogram of Oriented Gradients-based “get_frontal_face_detector()” and “shape_predictor()” functions are used. Isolated images of the eyes detected through dlib and captured through OpenCV are sent to a convolutional neural network (CNN) for gaze prediction. The CNN model was created through Keras and takes a 48 × 48 image of the eye as input and outputs the predicted zone a user gaze at. It was trained by capturing 50 images of the right eye of 4 users gazing at a total of 7 zones as well as blinking. This adds up to 400 images per user or 1600 images in total. The CNN architecture is summarized in Fig. 4. The predictions include center (C), left (L), right (R), top (T), bottom (B), lower left (LL), lower right (LR), and also blinking. Predictions are tied to conditional statements which determine the specific command to be executed.

For communication with the RPi4, the gpiozero library is utilized. This library allows for the remote control of RPi GPIO pins over WiFi. All pertinent files relating to the control system software are included in the database listed under Table 4 and are published under the Creative Commons License.

### 2.3 Capabilities and features

The technical specifications of MoMa are listed in Table 1. The design of MoMa enables it to be:

● Modular – To have space and separated parts that may be modified, reconfigured, and customized to fit specific needs
● Remotely controlled through gaze – To allow control of the robot only with eye positioning through the developed gaze prediction model, GUI, and interface with the controller
● Assistive – For users to be able to retrieve household objects, reaching shelves or tables as high as 75 cm
● Low-cost and open-source – To allow affordability for target users and researchers, and ease of access for assistive use, development purposes, or other robotic applications

## 3 Design files summary

There are three categories that make up the mobile manipulator design files, (a) the mechanical parts files, (b) the drivers, motors, and controllers files, and (c) the software files. Table 2, Table 3, Table 4 list down and describe all the pertinent files for each category. Table 2 describes all the mechanical parts used in constructing the mobile manipulator. These include the 3D CAD models of the 3D printed plates of the manipulator and the laser cut acrylic plates for the mobile base which were modeled using Onshape software. It also includes the 3D CAD models of other mechanical components such as wheels, couplings, bearings, screws and other accessories. Table 3 lists all the drivers, motors, and controllers used along with all the other electronic components. These include the DC motor, stepper motor, their motor drivers, the RPi, wires, batteries, and other accessories. Table 4 lists all the software files for the gaze control system. All the design files can be accessed through our repository: https://doi.org/10.17632/k7yfn6wdv7.1.

**Table 2:** Mechanical parts.

File No.	Description	File Type	License	Location of the File
Q1	Bottom Acrylic Plate	.stl	CC BY 4.0	Mendeley Database
Q2	Top Acrylic Plate	.stl	CC BY 4.0	Mendeley Database
Q3	Motor Plate	.stl (3D Print)	CC BY 4.0	Mendeley Database
Q4	Motor Connector Plate	.stl (3D Print)	CC BY 4.0	Mendeley Database
Q5	Gripper Plate	.stl (3D Print)	CC BY 4.0	Mendeley Database
Q6	Central Plate	.stl (3D Print)	CC BY 4.0	Mendeley Database
Q7	12 V Gear Motor Mount	.stl (3D Print)	CC BY 4.0	Mendeley Database
Q8	Mobile Manipulator Full Assembly	.stl	CC BY 4.0	Mendeley Database
P1	10 mm Rigid Flange Coupling	.step	CC BY 4.0	Mendeley Database
P2	12 mm Rigid Flange Coupling	.step	CC BY 4.0	Mendeley Database
P3	5 mm-12 mm Hex Coupling	.step	CC BY 4.0	Mendeley Database
P4	Ball Caster Wheel	.step	CC BY 4.0	Mendeley Database
P5	85 mm Wheel	.step	CC BY 4.0	Mendeley Database
P6	T10 Lead Screw (500 mm)	.step	CC BY 4.0	Mendeley Database
P7	T10 Lead Screw (850 mm)	.step	CC BY 4.0	Mendeley Database
P8	T10 Nut (2 mm lead)	.step	CC BY 4.0	Mendeley Database
P9	T10 Nut (4 mm lead)	.step	CC BY 4.0	Mendeley Database
P10	5 mm to 10 mm Flexible Aluminum Coupling	.step	CC BY 4.0	Mendeley Database
P11	6 mm Steel Rod (500 mm)	.step	CC BY 4.0	Mendeley Database
P12	6 mm Steel Rod (800 mm)	.step	CC BY 4.0	Mendeley Database
P13	SCS6UU Linear Ball Bearing	.step	CC BY 4.0	Mendeley Database
P14	KFL000 Flange Bearing	.step	CC BY 4.0	Mendeley Database
P15	6 mm Rigid Flange Coupling	.step	CC BY 4.0	Mendeley Database
P16	Brass Stud (65 mm)	.step	CC BY 4.0	Mendeley Database
P17	Brass Stud (60 mm)	.step	CC BY 4.0	Mendeley Database
P18	Brass stud (45 mm)	.step	CC BY 4.0	Mendeley Database
P19	Brass stud (25 mm)	.step	CC BY 4.0	Mendeley Database
P20	Brass stud (10 mm)	.step	CC BY 4.0	Mendeley Database
P21	3 mm Washer	.step	CC BY 4.0	Mendeley Database
P22	M3 Screw (10 mm)	.step	CC BY 4.0	Mendeley Database
P23	M3 Screw (15 mm)	.step	CC BY 4.0	Mendeley Database
P24	M3 Screw (45 mm)	.step	CC BY 4.0	Mendeley Database
P25	M3 Screw (50 mm)	.step	CC BY 4.0	Mendeley Database
P26	M4 Screw (10 mm)	.step	CC BY 4.0	Mendeley Database
P27	M4 Screw (45 mm)	.step	CC BY 4.0	Mendeley Database
P28	M6 Screw (20 mm)	.step	CC BY 4.0	Mendeley Database
P29	M6 Screw (25 mm)	.step	CC BY 4.0	Mendeley Database
P30	M3 nut	.step	CC BY 4.0	Mendeley Database
P31	M4 nut	.step	CC BY 4.0	Mendeley Database
P32	M6 nut	.step	CC BY 4.0	Mendeley Database
P33	Raspberry Pi Camera Mount	.png	CC BY 4.0	Mendeley Database
P34	VEX Claw Gripper	.step	CC BY 4.0	Mendeley Database

**Table 3:** Drivers, motors, and controllers.

File No.	Description	File Type	License	Location of the File
R1	12 V Gear Motor w/ Encoder	.png	CC BY 4.0	Mendeley Database
R2	NEMA17 Stepper Motor	.step	CC BY 4.0	Mendeley Database
R3	RPi 4	.png	CC BY 4.0	Mendeley Database
R4	BTS7960 Motor Driver	.png	CC BY 4.0	Mendeley Database
R5	2 s LiOn Battery (1000 mAh)	.png	CC BY 4.0	Mendeley Database
R6	3 s LiPo Battery (5600 mAh)	.png	CC BY 4.0	Mendeley Database
R7	Power Bank	.png	CC BY 4.0	Mendeley Database
R8	DRV8825 Motor Driver	.png	CC BY 4.0	Mendeley Database
R9	DRV8825 Expansion Board	.png	CC BY 4.0	Mendeley Database
R10	20 cm FF Jumper Wires	.png	CC BY 4.0	Mendeley Database
R11	20 cm MM Jumper Wires	.png	CC BY 4.0	Mendeley Database
R12	22AWG Wire	.png	CC BY 4.0	Mendeley Database
R13	Male XT60 to 14AWG wire	.png	CC BY 4.0	Mendeley Database
R14	XT60 Y Splitters	.png	CC BY 4.0	Mendeley Database
R15	XT60 F to Male T Plug Adapter	.png	CC BY 4.0	Mendeley Database
R16	RPi Camera	.png	CC BY 4.0	Mendeley Database
R17	RPi Camera Cable	.png	CC BY 4.0	Mendeley Database
R18	Logitech C920	.png	CC BY 4.0	Mendeley Database
R19	Rocker Switch	.png	CC BY 4.0	Mendeley Database

**Table 4:** Software files.

File No.	File Name	Description	File type	License	Location of the file
S1	gaze_control.py	Gaze control system	.py	CC BY 4.0	Mendeley Database
S2	training_model.py	Training and calibration of model	.py	CC BY 4.0	Mendeley Database
S3	shape_predictor.dat	Facial landmark detection	.dat	CC BY 4.0	Mendeley Database
S4	cnn	Neural network	folder	CC BY 4.0	Mendeley Database

### 3.1 Mechanical parts

The mechanical parts include the 3D CAD models of the 3D printed plates, the laser cut acrylic plates, and other components that make up the whole mobile manipulator, all summarized in Table 2. The parts are labeled as *P_n_* and *Q_n_* with *n* = 1, 2… where the label *Q* is for all the fabricated parts (3D printing, laser cutting) and the label *P* is for all other mechanical parts that make up the full assembly.

● *Q1* and *Q2* are the acrylic plates for the mobile base, where *Q1* houses the electronic parts and *Q2* houses the manipulator.
● *Q3-Q6* are the plates that attach to the mechanical components that make up the arms of the manipulator.
● *Q7* is the mount for the motor of the mobile base, where it is attached to the underside of *Q1*.
● *Q8* is the completed assembly of the mobile manipulator.
● *P1-P15* are used in fully constructing the mobile manipulator.
● *P16-P32* are washers, studs and screws that were used throughout the construction of the mobile manipulator.
● *P33* is the mount to connect the RPi camera to the manipulator.
● *P34* is the claw gripper taken from the VEX Clawbot which had an accompanying 2-wire motor 393 and a 3-wire motor controller 29.
● *P3, P10, P13-P15,* and *P34* are models that were acquired from the GrabCAD Library [7], [8], [9], [10], [11], [12].

### 3.2 Drivers, motors, and controllers

Table 3 summarizes all the drivers, motors, and controllers used in developing the mobile manipulator and the gaze control system. *R1-R19* are all the components listed for this section.

● *R1* and *R2* are the DC motors and stepper motors used in the mobile manipulator, respectively.
● *R3* allows for wireless communication to the gaze control system program and connects to all the motors and other electronic components.
● *R4* allows the DC motors to be controlled through PWM.
● *R8* and *R9* allows the stepper motors to be controlled through its number of steps and direction of rotation.
● *R5-R7* delivers the appropriate power for the motors and the RPi 4.
● *R10* and *R11* connect the motor drivers and other components to the RPi 4.
● *R12* extends the wires of the motor controller 29 to connect to the motor 393 of the gripper.
● *R13-R15* allows the batteries to connect to the motor drivers to power the motors.
● *R16* provides a live video feed of what is in front of the mobile manipulator which is displayed in the monitor for the gaze control system.
● *R17* extends the wire connection of the RPi camera to the RPi 4.
● *R18* is responsible for tracking the face and eye movement of the user for the gaze control system.
● *R19* is the emergency stop button which disconnects the batteries of the mobile manipulator to stop all the motors.

### 3.3 Software files

Table 4 outlines the pertinent software files for the developed system.

● *S1* is the main file for control of the system. This Python file interfaces the webcam, the host computer, and the RPi4. Additionally, it is responsible for all computer vision applications including detecting and capturing images of the eye and predicting location of the gaze as well as displaying the GUI.
● *S2* is a Supplementary file that allows users to train and calibrate their own CNN model. Instructions for use can be found in Section 6.4.
● *S3* is the facial landmark detector from dlib trained from the iBUG dataset.
● *S4* is the convolutional neural network used for gaze prediction

## 4 Bill of materials summary

Table 5 provides a summary of the bill of materials. Among the components listed in the table, the gear motor with encoder, RPi 4, RPi camera are sourced from the laboratory. The gripper itself is taken from an existing robotic kit. Note that the costing shown in the table does not include other extra costs (labor and transport). Additionally, 65 mm (P16) and 45 mm (P18) studs were not available during purchasing, so 20 mm extension studs were used in conjunction with 25 mm studs to match the lengths.

**Table 5:** Bill of materials.

Designator	Component	No.	Unit Cost (Php)	Total cost (Php)	Source of materials	Material type
P1	10 mm Rigid Flange Coupling	2	59	118	Link	Metal
P2	12 mm Rigid Flange Coupling	2	61	122	Link	Metal
P3	5 to 12 mm Hex Coupling (Pack of 4)	1	130	130	Link	Metal
P4	Ball Caster Wheels	2	80	160	Link	Others
P5	85 mm Wheels	2	150	300	Link	Other
P6	T10 Lead Screw (500 mm)	1	500	500	Link	Metal
P7-P8	T10 Lead Screw with Nut (850 mm)	1	670.40	670.40	Link	Metal
P9	T10 Nut (4 mm lead)	1	150	150	Link	Metal
P10	5 to 10 mm Flexible Aluminum Coupling	2	55	110	Link	Metal
P11	6 mm Steel Rods (500 mm)	2	216	432	Link	Metal
P12	6 mm Steel Rods (800 mm)	2	346	692	Link	Metal
P13	SCS6UU Linear Ball Bearing	4	190	760	Link	Other
P14	KFL000 Flange Bearing	2	84	168	Link	Other
P15	6 mm Rigid Flange Coupling	8	56	448	Link	Metal
P16, P18	20 mm Brass Stud Extensions	10	6	60	Link	Metal
P17	60 mm Brass Studs (Pack of 10)	1	155	155	Link	Metal
P19	25 mm Brass Studs (Pack of 10)	1	55	55	Link	Metal
P20	10 mm Brass Studs (Pack of 10)	1	30	30	Link	Metal
P21	3 mm Washers (Pack of 50)	1	50	50	Link	Metal
P22	M3 Screws (10 mm)	80	0.20	16	Hardware store	Metal
P23	M3 Screws (15 mm)	36	0.30	10.8	Hardware store	Metal
P24	M3 Screws (45 mm)	16	0.40	6.4	Hardware store	Metal
P25	M3 Screws (50 mm)	8	0.40	3.2	Hardware store	Metal
P26	M4 Screws (10 mm)	8	0.40	3.2	Hardware store	Metal
P27	M4 Screws (45 mm)	4	0.80	3.2	Hardware store	Metal
P28	M6 Screws (20 mm)	2	1	2	Hardware store	Metal
P29	M6 Screws (25 mm)	2	1	2	Hardware store	Metal
P30	M3 Nuts	80	0.30	24	Hardware store	Metal
P31	M4 Nuts	4	0.30	1.2	Hardware store	Metal
P32	M6 Nuts	4	0.50	2	Hardware store	Metal
P33	RPi Camera Mount	1	77	77	Link	Polymer
P34	VEX Claw Gripper	1	−	−	−	Other
R1	12 V Gear Motor w/ Encoder	2	5209	10,418	Link	Other
R2	NEMA17 Stepper Motor (42 × 42 × 34)	2	355	710	Link	Other
R3	RPi 4	1	2156	3099	Link	Other
R4	BTS7960 Motor Driver	2	416.5	833	Link	Other
R5	2 s LiOn Battery (1000 mAh)	1	659	659	Link	Other
R6	3 s LiPo Battery (5600 mAh)	1	1880	1880	Link	Composite
R7	Power Bank	1	669	669	Link	Other
R8	DRV8825 Motor Driver	2	169	338	Link	Other
R9	DRV8825 Expansion Board	2	130	260	Link	Other
R10	20 cm FF Jumper Wires (Pack of 40)	1	65	65	Link	Other
R11	20 cm MM Jumper Wires (Pack of 40)	1	65	65	Link	Other
R12	22AWG Wire (20 m, Pack of 4)	1	90	90	Link	Other
R13	Male XT60 to 14AWG wire	5	60	300	Link	Other
R14	XT60 Y Splitters (2F to 1 M)	3	77	231	Link	Other
R15	XT60 F to Male T Plug Adapter	1	64.75	64.75	Link	Other
R16	RPi Camera	1	2347	2347	Link	Other
R17	RPi Camera Cable (2 m)	1	179	179	Link	Other
R18	Logitech C920	1	4975	4975	Link	Other
R19	Rocker Switch	1	15	15	Link	Other
Q1-Q2	Acrylic Plates	2	1350	2700	Fabricated/Laser cut	Polymer

## 5 Build instructions

The parts and materials listed in Table 2 and Table 3 must first be acquired. The acrylic plates must be cut, and the 3D printed parts printed according to the dimensions as specified in the files found in Table 2. Once all the materials are available, the assembly may begin. Presented in this section are instructions that should be followed sequentially to construct the robot. Before starting the assembly, some tools and materials must be prepared, including lubricating oil, a screwdriver, and a hex wrench.

### 5.1 Hardware build instructions

The building of the mobile base may be done first in order to ensure safe and efficient assembly. The mobile base may later serve as a platform for the arm to be attached with stability. Additionally, as the RPi 4, batteries, and drivers will be installed on the mobile base, it will be easier to access these components for proper wiring and connecting, as well as testing, before the entire robot is assembled. In these instructions, the diameter and length of the screws will be specified, for example as “M3 × 10”, which refers to an M3 size screw with a 10 mm length. Make sure to use the appropriate size of nuts and washers where necessary. Detailed below are instructions in assembling the robot:

1. Motor assembly: The parts required for this step are the two (2) motor mounts (Q7), eight (8) M4 × 10 screws (P26), two (2) of the 10 mm rigid flange couplings (P1), two (2) of the 12 mm rigid flange couplings (P2), two (2) of the 5 to 12 mm hex couplings (P3), two (2) 85 mm wheels (P5), and eight (8) M3 × 10 screws (P22). In this step, two motor assemblies will be completed. Fasten a motor to a mount using 4 of the M4 × 10 screws. Then, fasten a 10 mm rigid flange coupling to the shaft of the motor by using the hex wrench to tighten the screws in the flange coupling. Using four (4) M3 × 10 screws, attach a 12 mm rigid flange coupling facing opposite the 10 mm rigid flange. Then, insert one 5 mm-12 mm hex coupling to the other side of the 12 mm rigid flange coupling and fasten it. Each motor assembly should appear as shown in Fig. 5a. Then, fasten a wheel on the end of the hex coupling with the accompanying screw. The final motor assembly appears as shown Fig. 5b. Repeat to complete another motor assembly.

**Fig. 5:**
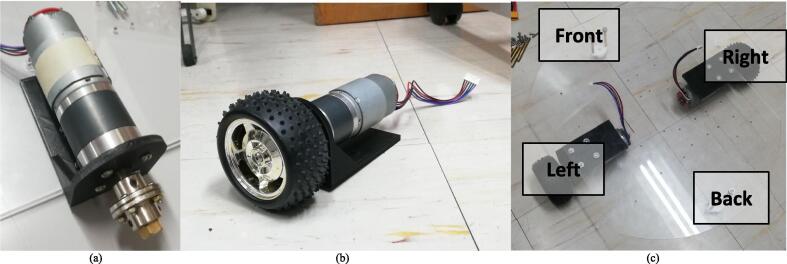
(a) The motor assembly with the rigid coupling assembly attached. (b) The complete motor assembly. (c) Bottom base assembly.
2. Bottom base assembly: The parts required for this step are the bottom acrylic plate (Q1), the two (2) ball caster wheels (P4), two (2) 45 mm brass studs (P18), four (4) M3 × 10 screws (P22), eight (8) M3 × 15 screws (P23), and 12 3 mm washers (P21). Due to lack of stock, extensions were used to create the 45 mm brass studs; fasten one 20 mm brass stud extension to each leg of the two ball caster wheels. Then, the motor assemblies completed in the previous step and the caster wheel assemblies can be attached to the bottom acrylic plate. Fasten one caster wheel assembly near the front and one near the back of the acrylic plate using the M3 × 10 screws, and then one motor assembly on the right and one on the left of the plate using the M3 × 15 screws. Use washers where the screw meets the plate. The result is shown in Fig. 5c.
3. Electronic components and studs for top acrylic plate: The two (2) BTS7960 Motor Drivers (R4) and its accompanying boards, and two (2) DRV8825 Motor Drivers (R8) mounted on their two (2) Expansion Boards (R9), the 3 s LiPo Battery (5600 mAh) (R6), the RPi 4 (R3), and the power bank (R7) will now be fixed on the base. Use four (4) 25 mm studs (P19) to elevate each of the BTS7960 drivers from the bottom acrylic plate, four (4) 10 mm studs (P20) for the DRV8825 drivers. A total of 32 M3 × 10 screws are needed to fasten the four drivers. Then, fasten the RPi 4 with M3 screws and nuts. The LiPo battery and the power bank may be fastened to the base using velcro strips. Then, attach 60 mm studs (P17) around the bottom acrylic plate, using M3 × 15 screws for each stud. These studs will support the top acrylic plate later on. So far, the layout of the components on the base should look as shown in Fig. 6. The 2S 1000 mAh LiPo battery (R5) is also placed on the bottom base and may be secured with velcro strips as well. By the end of this step, the making of the necessary wirings and connections as detailed in the following Section 5.2 may be started.

**Fig. 6:**
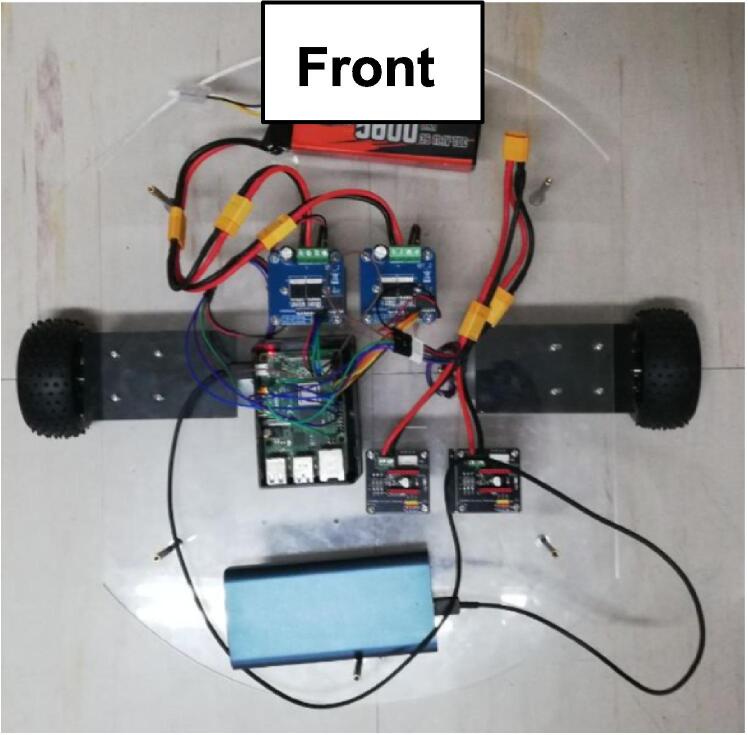
Bottom base assembly with electronics, batteries, and studs. The front of the robot is labeled.

The following items are instructions to build the arm of the robot. Before assembling the arm, it is recommended to lubricate the T10 nuts and the lead screws with oil before attaching them to the plate, to improve the performance and ensure smooth operation of the system.

4. Central plate assembly: Attach two (2) linear bearings (P13) (using 4 × M3 × 45 screws each, P24) and the 2 mm lead T10 traveling nut (P8) (using 4 × M3 × 50 screws each) to the vertical side of the central plate, as shown in Fig. 7a. On the horizontal side of the plate, attach two linear bearings as well. Use two 4 mm lead T10 nuts (P9) for this side, as shown in Fig. 7b.

**Fig. 7:**
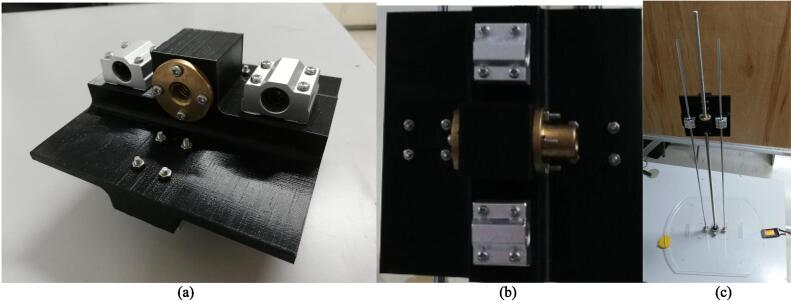
Side of the central plate that holds (a) the vertical rods and (b) the horizontal rods. (c) Top base assembly including the vertical lead screw and rods.
5. Top base assembly: Attach two (2) 6 mm rigid flange couplings (P15) (4 × M3 × 10 each) and the flange bearing (P14) (2 × M7 × 25 each) to the top base. Attach the T10 × 850mm lead screw (P7) to the flange bearing by tightening the hex screws. Attach the 800 mm support rods (P12) to the rigid flanges. Use 1 M3 × 10 screw to tighten each of the support rods to the rigid flange; however, if available, shorter M2.5 screws would be more suitable. Then, attach the central plate assembly to the screw and rods. Do so by securing the base and rotating the screw to lower the plate. The top base assembly must appear as shown in Fig. 7c.
6. Top motor assembly: Attach two 6 mm rigid flanges (P15) (4 × M3 × 10 each) to a motor plate (Q3), as shown in Fig. 8a. Then, attach the stepper motor (R2) (4 × M3 × 10) to the motor connector plate. Attach the flexible coupling (P10) to the motor shaft by tightening the hex screws. Attach the motor connector plate (Q4) to the top plate using four (4) 60 mm-long studs (P17) (2 × M3 × 15 each), as shown in Fig. 8b. Then, fix the lead screw to the flexible coupling, and fasten the support rods (1 × M3 × 10 each) to the rigid flanges of the top plate. The result is shown in Fig. 8c.

**Fig. 8:**
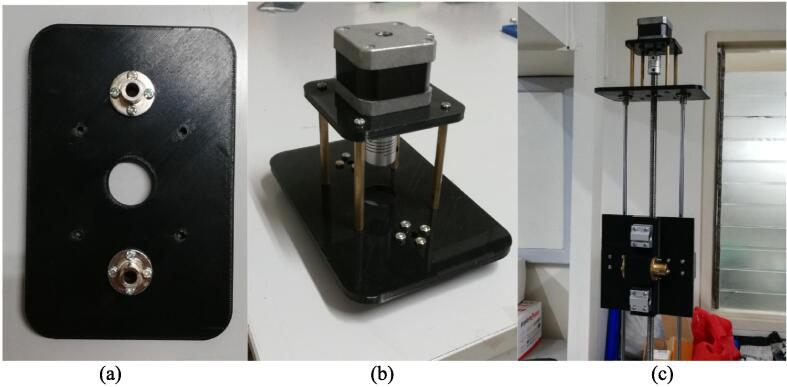
(a) Motor plate with rigid flanges. (b) Top motor assembly (c) mounted.
1. Side motor assembly: Follow the same procedure for attaching the motor in the top motor assembly, this time using 25 mm studs (P19). The side motor assembly looks as shown in Fig. 9a.

**Fig. 9:**
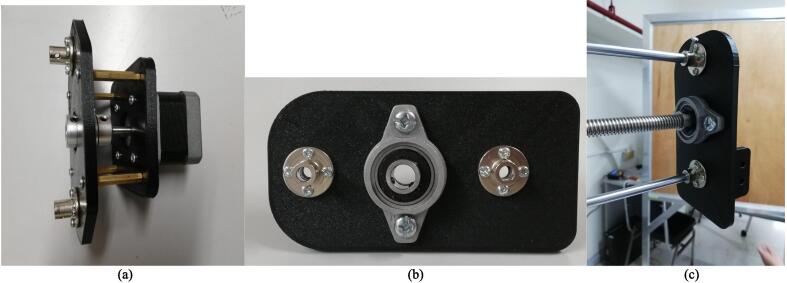
(a) Side motor assembly. (b) Gripper plate assembly (c) mounted.
2. Gripper plate assembly: Attach two (2) 6 mm rigid flanges (P15) (4 × M3 × 10 each) and a flange bearing (P14) (2 × M6 × 20, P28) to the gripper plate, using the appropriate nuts (P30, P32). The result is shown in Fig. 9b. Attach the gripper plate to the two (2) 500 mm support rods (P11) to the rigid flanges (using an M3 × 10 each), and the T10 × 500mm lead screw (P6) to the flange bearing, as shown in Fig. 9c.
3. Completing the horizontal linkage of the arm: Fit the horizontal support rods into the horizontal linear bearings, and the horizontal lead screw into the nut. Then, attach the side motor assembly. Fix the support rods into the rigid flanges (1 × M3 × 10 each) and the lead screw into the flange bearing. Attach the gripper to the gripper plate (using 4 × M4 × 45, P27), as shown in Fig. 10a.

**Fig. 10:**
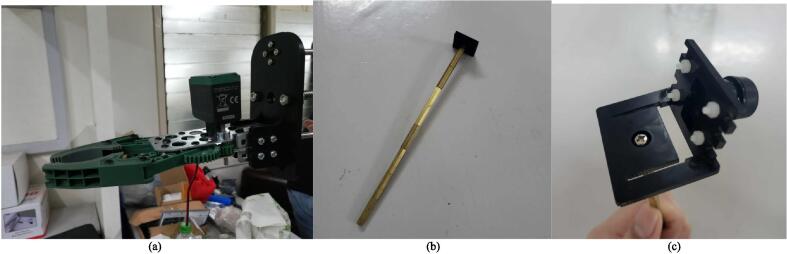
(a) The gripper mounted on the gripper plate. (b). The studs used as a stand for the camera mount. (c) The camera assembly with the camera mounted.
4. RPi Camera assembly: Use a 65 mm stud (P16) and attach it to the camera mount assembly as shown in Fig. 10 b and c. Note that due to availability of materials, a different number of studs were used and connected together instead. The overall assembly is presented in (Fig. 11).

**Fig. 11:**
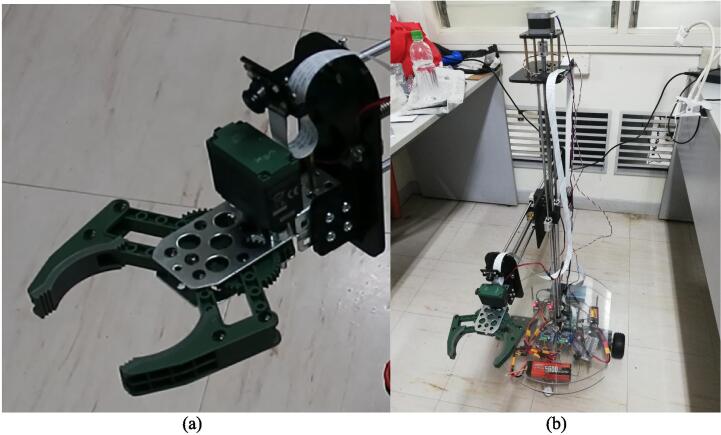
(a) The gripper mounted on the gripper plate. (b). The complete assembly.

### 5.2 Wiring and connections

**Fig. 12:**
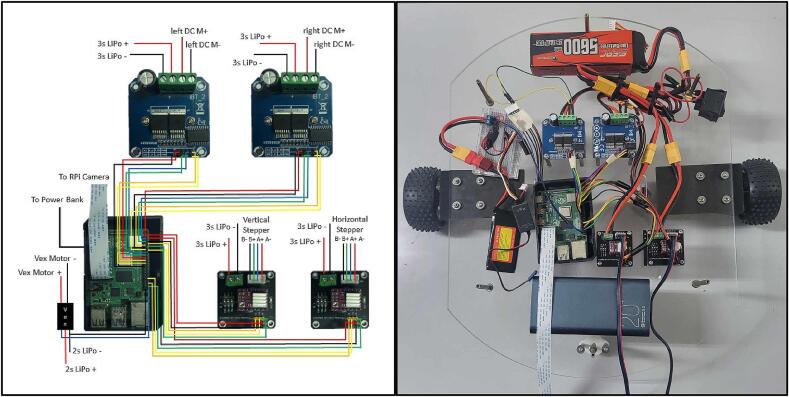
(a) Complete schematic diagram for connections, (b) and actual connections.

The complete assembly of the mobile manipulator involves specific wirings and connections to ensure that a) the RPi4 is able to properly control components and receive information, and b) the motors receive sufficient power. A summarized schematic of all the connections as well as actual photos are shown in Fig. 12.

1. Ensure that all the drivers are secured as instructed in Section 5.1. Before wiring making and driver connections, ensure that the drivers are not powered.
2. Begin by wiring the BTS7960 drivers (R4) to the RPi4 (R3) using the 20 cm female-to-female breadboard jumpers (R10). Connections labels are based on those printed onto the PCB layout of the drivers. Specific connections to the RPi4 GPIO pins are outlined in Table 6.

**Table 6:** Summary of pin connections.

Driver	Driver Pin	GPIO Name (Pin #)	Driver	Driver Pin	GPIO Name (Pin #)
BTS7960 (Right)	VCC	3v3 (Pin 1)	DRV8825 (Horizontal)	VCC	5 V (Pin 2)
	GND	Ground (Pin 9)		GND	Ground (Pin 6)
	R_EN	GPIO2 (Pin 3)		EN	GPIO14 (Pin 8)
	L_EN	GPIO3 (Pin 5)		DIR	GPIO15 (Pin 10)
	RPWM	GPIO4 (Pin 7)		STEP	GPIO18 (Pin 12)
	LPWM	GPIO17 (Pin 11)	DRV8825 (Vertical)	VCC	5 V (Pin 4)

BTS7960 (Left)	VCC	3v3 (Pin 17)		GND	Ground (Pin 14)
	GND	Ground (Pin 25)		EN	GPIO16 (Pin 36)
	R_EN	GPIO27 (Pin 13)		DIR	GPIO20 (Pin 38)
	L_EN	GPIO22 (Pin 15)		STEP	GPIO12 (Pin 32)
	RPWM	GPIO10 (Pin 19)	VEX Motor Controller 29	PWM	GPIO21 (Pin 40)
	LPWM	GPIO09 (Pin 21)		GND	Ground (Pin 39)
3. Connect the two DRV8825 (R8-9) drivers to the RPi4 using the 20 cm female-to-female breadboard jumpers. The connections to the RPi4 GPIO pins are outlined in Table 6.
4. Connect the VEX motor driver (P34) to the RPi4 based on the connections outlined in Table 6 using the 20 cm female-to-female breadboard jumper wires. Ensure that the wire connecting to the RPi4 wire is able to run in parallel with another wire, either through a breadboard or through soldering another connection.
5. Once all the drivers have been connected to the RPi4, the motors can now be connected to their respective drivers. Connections are made by loosening the screws at each terminal, inserting the wires, then tightening the screws. The DC Motors (R1) can be connected to both BTS7960 motors using the 20 cm male-to-male jumpers (R11) as labeled by the PCB layout and shown in Fig. 12a. Ensure that the motor attached to the left of the base is connected to the left driver, and the right motor to the right driver. Stepper motors (R2) can be connected to the DRV8825 hats using the included 4-wire cables as shown in Fig. 12a. Lastly, the VEX motor attached to the gripper can be connected to the motor controller 29 by soldering around 2.5 m of wire (R12). Ensure that all connections follow the proper terminals.
6. For safety, one may opt to solder a switch (R19) to the two LiPo batteries. With the switch turned OFF, connect the battery to the two BTS7960s and the two DRV8825s. Parallel connections can be made through the Y splitters (R14) to the 3 s battery (R6) as shown in Fig. 13.

**Fig. 13:**
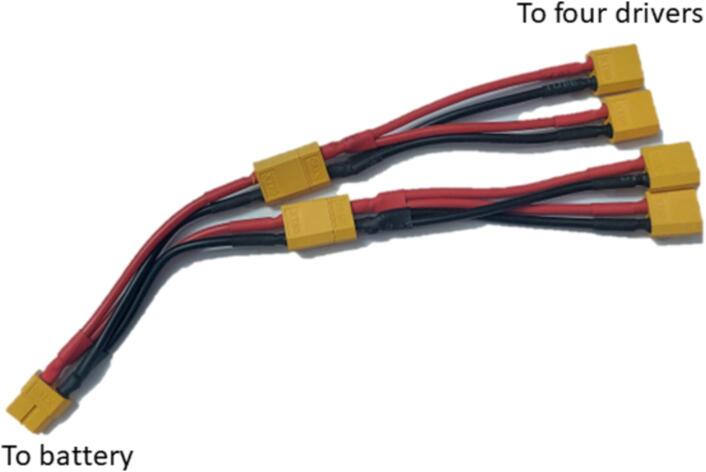
Y Splitter connections.
7. The 2S 1000 mAh LiPo battery (R5) can also be connected to the VEX motor controller. The output port of the 2S battery is a Dean’s T Plug, so the T Plug adapter (R15) must be used.
8. To power the RPi, the 20000 mAh power bank (R7) can be connected via a USB-C cable. Leave the RPi disconnected when not in operation.
9. With all the drivers, motors, and power sources connected, the last step is to connect the RPi4 to the camera through the 2 m ribbon cable (R17).

## 6 Operation instructions

This section outlines the operation of the mobile manipulator with the gaze control system. It includes guidelines on setting up both the RPi and the host computer, interfacing, controlling, and also using the included training software.

### 6.1 Setting up the RPi

In order to operate the system, it is essential to set up the RPi4 with the appropriate settings and libraries as well as prepare it for interfacing with the host computer.

1. Connect the RPi4 to a monitor, keyboard, and mouse. Then, power up the RPi4.
2. Ensure that the RPi4 is connected to the same wireless network as your host computer. Interfacing will not be possible without this step. Once connected, get the IP of the RPi4 opening the command line and typing in the following command. Note down the output.

**Figure:**
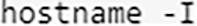

3. Using the command line, update and reboot the RPi4 by executing the first two commands shown in Fig. 14. This ensures that all existing libraries are up to date. Then, install and enable pigpio and gpiozero through the next three lines shown in the same figure.

**Fig. 14:**
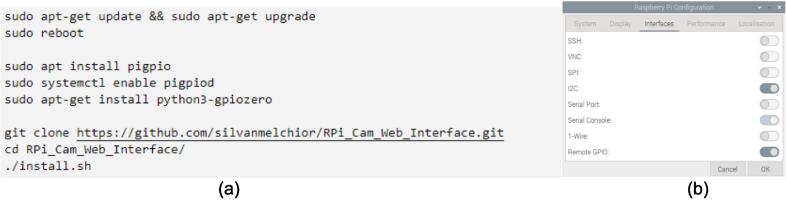
Setting up the RPi4 by (a) installing libraries and (b) turning on remote GPIO.
4. Install the wireless interface for the RPi camera. For this application, RPI Cam Web Interface developed by Silvan Melchior is used [13]. Clone the repository using the git clone command. After downloading, enter the folder using the cd command, then install the repository.
5. The next step is to enable Remote GPIO in order to allow external wireless devices to control the RPi’s pins. Open the RPi4 configuration settings, click on the Interfaces tab and enable Remote GPIO. This is also shown in Fig. 14.
6. The setup is complete, the RPi4 may be shut off, disconnected, and placed on the robot base.

### 6.2 Setting up the host computer

After setting up the RPi4, the host computer must be set up to properly execute the code and interface with the RPi4.

1. After booting up the computer, install the following libraries by entering the following commands in the Windows command Prompt. Note that Python [14] must already be installed on the computer. In order to properly install dlib, both cmake [15] and visual Studio [16] must also be installed.

**Figure:**
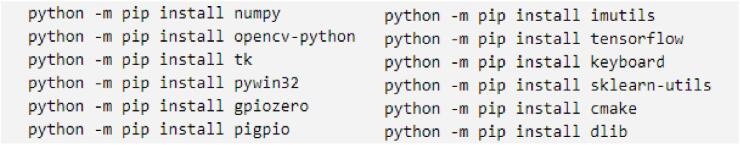

2. After installing the appropriate libraries, open gaze_control.py (S1) through an IDE or text editor. The code must be edited to contain the proper information and file paths. The specific lines of code are shown below.

**Figure:**
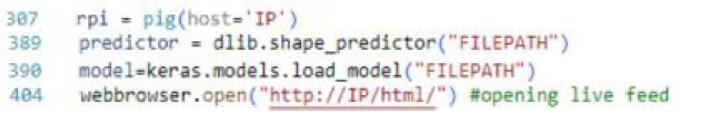

In line 307 and 404, replace the word IP with the IP address of the RPi4. In line 389, replace the word FILEPATH with the file path where shape_predictor.dat (S3) is stored. Lastly, in line 390, replace the word FILEPATH with the file path where cnn (S4) or a self-calibrated model is stored. (Note that Windows file paths use backslashes (\) as delineators, while Python requires forward slashes (/). Be sure to replace all forward slashes.)

3. After preparing the code, connect the webcam to the host computer. When using a laptop with the external webcam, the code may default to using the laptop’s built-in webcam. If this occurs, change the 0 in line to 1.

**Figure:**
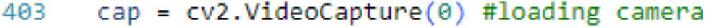

### 6.3 Interfacing and controlling the robot

The system is now ready to be interfaced and run. For safety, keep the mobile manipulator powered off until actual use to prevent accidental movements when initially running the code.

1. Power on the RPi4. Run the gaze_control.py code through the command prompt by executing the commands shown below. Replace FILEPATH with the designated file path for the folder containing the code.

**Figure:**
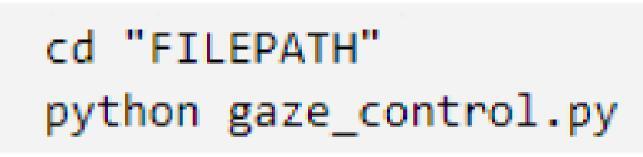

2. The code will begin running and interfacing with the RPi. If the error: *“OSError: Failed to connect to 192.xxx.xxx.xxx:8888”* appears, it could mean any of the following errors in setting up: a) the RPi or host computer is not connected to the WiFi, b) the RPi and the host computer are not connected to the same network, c) the given IP address is incorrect. Otherwise, the interfacing will occur automatically.
3. The code will then display the GUI and redirect the user to a webpage where the live feed is displayed as shown in Fig. 15. Double clicking on the feed will allow it to enter full screen.

**Fig. 15:**
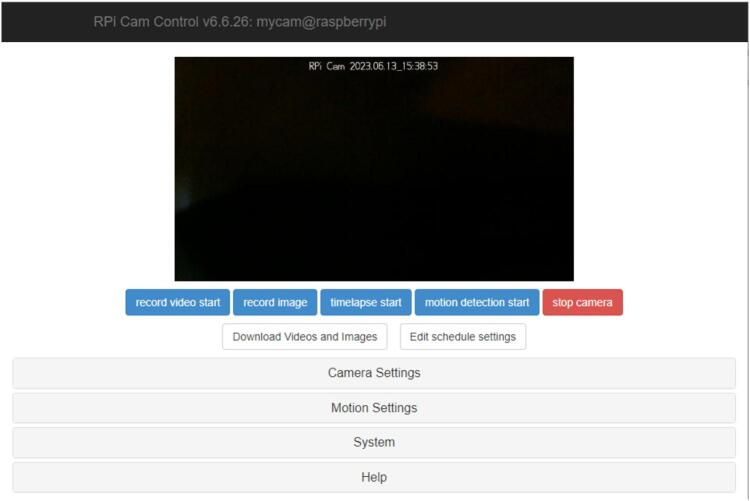
RPI webcam interface portal.
4. When the webcam boots up, the system is finally completely running. Users can practice activating each command and switching between mobile base and manipulator control while the mobile manipulator is turned off to ensure safety. The selected command will light up green (for base control) or red (for manipulator control) when activated (dwell time of 1 s).
5. When ready, the user can then turn on the mobile manipulator and fully control it through gaze. Fig. 3 can be used as a reference for the specific commands per zone.
6. When terminating the control of the robot, users must rest their eyes on the center of screen to ensure that the robot is stopped. Turn off the robot and press the ESC key to terminate the system.

### 6.4 Training your own model

In the event that the control system has difficulty in predicting the direction of gaze for a specific user, it may be necessary to train a model specific to the user. training_model.py (S2) can be used to train a neural network to predict the gaze of a specific user. The program takes 50 images of the user gazing at each specific zone as well as blinking for a total of 400 images.

1. Similar to the previous procedure, the python code must be edited before use. After opening training_model.py using a text editor or an IDE, the following lines must be edited. Line 171 must be edited to contain the file path of the shape_predictor.dat file. Lines 198 and 214 must be edited to contain the file path to a specific folder where the user wants to save the photos. Note that a / must be attached to the end of the folder file path for lines 198 and 214. Lastly, line 259 must be edited to have the desired file name for the CNN to be made.

**Figure:**
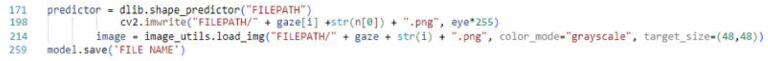

2. The code can be run through the command prompt by using the cd command with the file path of the folder, then running the code using the python command as shown below. Ensure that the webcam is plugged in before running.

**Figure:**
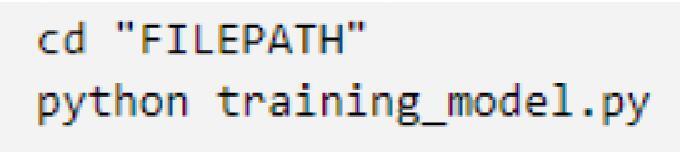

3. Running the code will result in seven zones being displayed on the screen. The photos showing the process of training are outlined by Fig. 16. The center zone that users must gaze at is marked red. Once users are at a comfortable position and have fixed their gaze on the red zone, pressing the spacebar will change the color to green, signifying that the system is now taking photos of the user’s eyes. Users must fix their gaze on the zone until it turns gray (approximately 20 s). This process continues for each of the 7 zones until images for all zones have been taken.

**Fig. 16:**
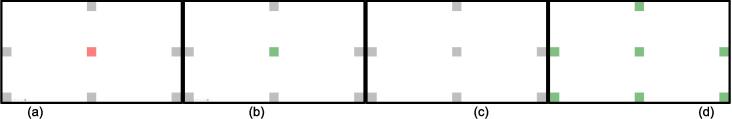
Images of the GUI for training when a) a zone is marked red, b) the system is recording the users staring at the zone, c) the system is ready to take photos of the user blinking, and d) the system is taking photos of the user blinking. (For interpretation of the references to color in this figure legend, the reader is referred to the web version of this article.)
4. All of the seven zones will then turn gray. At this point, the system is ready to take images of the user blinking. Users must close their right eye, then click the spacebar. All seven zones will light up green while taking photos of the user blinking.
5. Once each box turns gray once more, the GUI will then be closed, and the program will automatically prepare the image data, train, and save the neural network in the same folder. Users can now deploy the model.

## 7 Validation and characterization

The MoMa system is designed to utilize an accurate and responsive webcam-based gaze control system. It is also designed to handle pick and place tasks safely for objects specified in Section 2.1. This involves the mobile manipulator being able to safely maneuver through flat terrain at an appropriate speed, firmly grip the desired object, and safely transport it to a desired location. Testing was conducted to validate these design goals and to ensure its usability in real life applications, a video is uploaded at the Medeley Repository. The results of testing are presented in this section.

### 7.1 Testing accuracy of control system

**Fig. 17:**
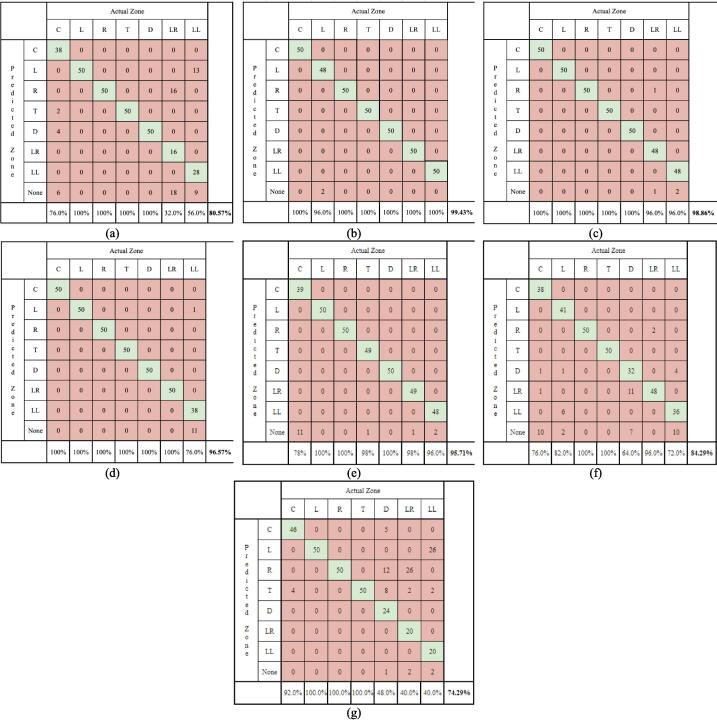
Confusions matrices for (a) participant 1, (b) participant 2, (c) participant 3, (d) participant 4, (e) participant 5, (f) participant 6, (g) participant 7.

In testing the accuracy of the control system, confusion matrices were utilized as a form of measurement and evaluation. Data is gathered by tasking participants to gaze at each of the seven zones a total of 50 times each. The developed model then creates its own inference on which zone was gazed at by the user after a 1 s dwell time. This prediction is then compared to the actual zone through the confusion matrix. The system was tested by a total of seven users. Four of the users (Users 1 to 4), were the same participants whose eyes were used to train and calibrate the system. The confusion matrices corresponding to these users are shown from (a) to (d) of Fig. 17. Three of the users (Users 5 to 7) did not have the system calibrated for their eyes. This allows for the results to give some insight on the overall usability and robustness of the system when utilized by users it is not calibrated for. The results for the latter three users are shown from (e) to (g) of Fig. 17.

**Fig. 18:**
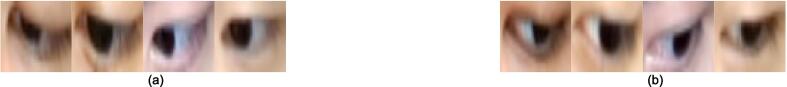
Images of the gazing at the (a) lower left for zone for participants 1 to 4 respectively, and (b lower right zone for participants 1 to 4 respectively.

The results show that the accuracy of the system for participants 1 to 4 are predominantly high with majority of the accuracy readings being above 96 %, however, for participant 1, it is observed that the system struggled to accurately predict his gaze. This is attributed to the shape and size of User 1′s eye. As shown in Fig. 18, given a particular movement, looking down, User 1′s eyelids droop low which obstructs the camera from detecting the iris, consequently, making it more difficult for the model to make predictions. This suggests that the system works more accurately when the eye is fully seen.

Overall, the system’s accuracy for users with calibration is nearly 94 %. A similar study by Mounica et al. [3] developed a webcam-based eye tracker with an accuracy of 95 %. Although the accuracy of MoMa’s control system is slightly lower than 95 %, it is important to note that the system is trained for four users rather than one user for the other study. Additionally, the developed system allows for prediction for seven zones across three rows while that of the other study only allows for six zones across two rows. As such, the developed system is deemed accurate for users with calibration.

For the three users without calibration, the accuracy of the system was found to be 95.71 %, 84.29 %, and 74.29 % respectively, and overall, the system’s accuracy for users without calibration is only 84.76 %. It is observed that the accuracies for the three users have more variation as compared to that of the previous four users. Additionally, the overall accuracy of the system is lower. This suggests that the system’s performance for users without calibration is unpredictable at that moment. While the system may function well without prior training, as evidenced by the accuracy for User 5, it may be more beneficial for users to utilize training_model.py to calibrate their own models for better accuracy. To increase the general reliability of system, more training data may be obtained and used to train the developed model.

### 7.2 Testing latency of control system

In terms of safety, the Mobile Manipulator is equipped with an emergency stop switch located on the side of the mobile base, directly connected to the power supply. This design allows users to immediately halt all motor activity in emergency situations. Additionally, the gaze-control system, which requires the user’s gaze to dwell on specific areas of the screen to initiate commands, ensures that the Mobile Manipulator can be safely stopped when the gaze is disengaged from these command zones. The traversing speed of the Mobile Manipulator is set at 15 cm/s, which will be discussed further in subsequent sections, as it is the recommended safe speed according to King et al. [21].

Latency is also considered in delivering the input commands from the user to the gaze-control system, then delivering that command to the actual motors of the robot to execute it. A system with low latency is responsive and safe for users as it can quickly carry out desired actions and immediately stop when users see the need to terminate motion. It is important to note that latency is different from dwell time. Dwell time is an intentional delay used to prevent the accidental triggering of commands. Latency is an undesirable delay caused by processing time and wireless communication. To test latency, the total time from when a frame is read until the command is executed by the RPi4 was recorded across 50 trials. The average latency is then taken and compared to the latency of existing commercial eye trackers utilized for robotic gaze control systems by previous studies [2], [17], [18], [19].

Table 7 summarizes the latency of different gaze estimation systems utilized in robotic applications.

**Table 7:** Comparison of system latencies.

Eye tracker	Latency
MoMa’s Gaze Control System	116 ms
EyeLink 1000 Plus	1.4 ms
Tobii 1750	25–35 ms
Gazepoint GP3	16.67 ms
Tobii PCEye 5	25 ms

The table reveals that the developed gaze control system exhibits significantly higher latency compared to commercial eye trackers. However, this increased latency in the webcam-based gaze control system has negligible effects on the specific application, both in terms of responsiveness and safety. This is largely due to the fact that typical dwell times for gaze control systems range from 0.5 to 2 s [19], [20], placing the system's performance well within acceptable limits.

With the system's programmed dwell time of 1 s and an average latency of 0.116 s, a command is triggered in approximately 1.116 s, which falls within an acceptable range for dwell-based control systems. This demonstrates the system’s responsiveness, comparable to other developed systems. In terms of safety, the latency has minimal impact due to the robot's low velocity of 15 cm/s. A 0.116-second delay results in only 1.74 cm of excess motion, ensuring that the robot can stop quickly and safely. These results indicate that the system’s latency is within acceptable limits for both responsiveness and safety.

### 7.3 Load calculations and stress simulations

In order to ensure that the construction and design of the mobile manipulator is safe, load and stress calculations were done and validated using Solidworks. The design calculations and simulations are summarized Table 8. Five components were tested, namely the 500 mm T10 lead screw, 500 mm support, 800 mm T10 lead screw, 800 mm support, and the central plate. All of them were tested and simulated for a 1 kg load. Considering the price and availability of materials, the corresponding materials were chosen. Based on this, the equivalent von Mises stress for each component was calculated along with their factor of safety. This was then validated through Solidworks stress simulations where it showed that the calculated stresses were close to the values obtained from the simulated stress results. All components resulted in factors of safety higher than 2 which is the recommended factor of safety for pick and place tasks for manipulators [21].

**Table 8:** Manipulator design summary.

Component	Material	Calculated Von Mises Stress	Factor of Safety	Simulated Von Mises Stress	Factor of Safety
T10 Screw (500 mm)	AISI 304	45.9283 MPa	4.6812	45.17 MPa	4.76
6 mm Support (500 mm)	AISI 52100	74.47 MPa	5.57	75.90 MPa	5.47
T10 Screw (800 mm)	AISI 304	32.48 MPa	6.62	35.53 MPa	6.05
6 mm Support (800 mm)	AISI 52100	75.207 MPa	5.52	82.48 MPa	5.03
Central Plate	PLA Filament	−	−	0.788 MPa	46.95

The tipping of the mobile manipulator system was evaluated. Onshape was used to determine the center of mass. After assigning all the masses of the components to the CAD models, the center of mass was obtained where it was found to be close to 350 mm from the ground and within or in the middle of all the four wheels of the mobile base. This means that under the load of all the components of the system, it is safe from tipping.

It is also important to ensure that the robot will not tip when carrying the designed load. Considering the weight of a 1 kg load for the arm fully extended, the resulting moment was found to be 1.962 Nm about the front wheel. The moment of the robot’s mass about this same point was found to be 14.526 Nm, which is far greater than the moment caused by the 1 kg load. This means that the robot will not tip under the 1 kg load even when fully extended.

### 7.4 Encoder and motor calibration

The mobile manipulator is designed to move at a speed of 15 cm/s along a straight path. In this study, MY-37 encoders, which are two-channel Hall effect encoders, were connected to each of the motors, one for the left wheel and one for the right wheel. Tests were then conducted in order to calibrate the encoders by determining the number of ticks that corresponded to the forward movement of the robot by one meter.

**Fig. 19:**
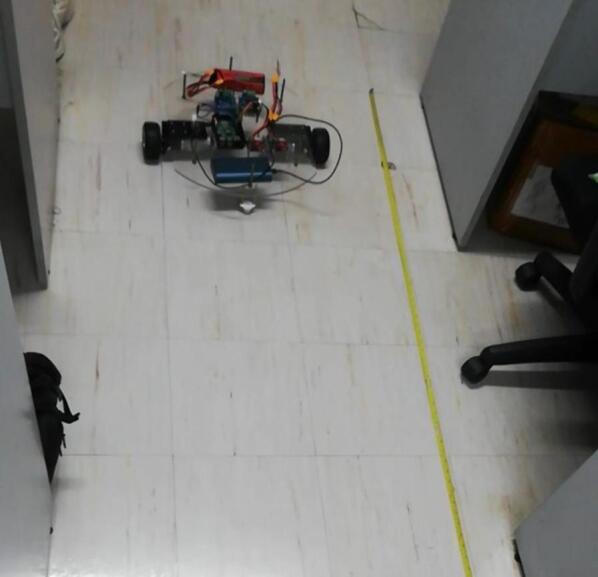
Encoder calibration test setup.

The motors were made to run using PWM at a frequency of 100 Hz and a 50 % duty cycle for 10 s along a straight path as shown in Fig. 19. At the end of 10 s, the motors were made to stop. The position of each wheel was then recorded using a tape measure, and the rotation of each wheel measured by the encoders in ticks were taken. Several sources of error may have been present in the testing, however, including human error when taking physical measurements, the slippage of the wheel due to the lack of traction and imperfections in the path, and instrument error. As such, 10 trials were conducted, and the average ticks per meter was taken. The ticks per meter were found to be 1291.86 for the right wheel, and 1292.66 for the left wheel. The results of this test are shown in Table 9.

**Table 9:** Encoder calibration test results.

Trial	Right		Left	
	Ticks	Distance (cm)	Ticks	Distance (cm)
1	2401	186.20	2334	180.00
2	2365	183.00	2313	179.40
3	2366	183.50	2308	179.00
4	2371	183.50	2309	178.50
5	2377	184.20	2306	178.70
6	2360	182.50	2302	178.00
7	2322	180.00	2287	177.20
8	2362	182.50	2287	177.20
9	2352	181.70	2285	176.00
10	2347	181.50	2268	175.20
**Average**	**2362.3**	**182.86**	**2299.9**	**177.92**

	**Ticks per meter**	**1291.86**	**Ticks per meter**	**1292.66**

**Fig. 20:**
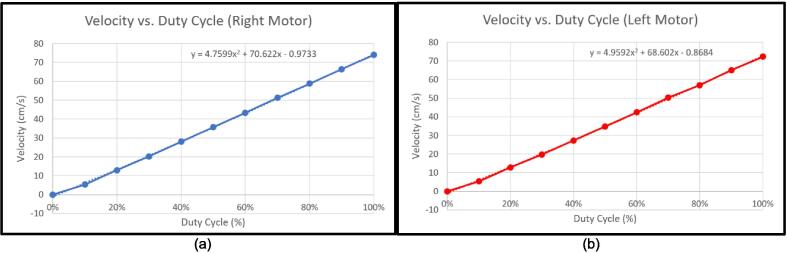
Velocities for different duty cycles for the (a) right motor, and the (b) left motor.

After establishing the number of ticks per meter, the motors were then calibrated for speed. From a 10 % duty cycle, incrementing upwards by 10 % until 100 %, the left and right motors were made to run for 5 s. The ticks were then measured and averaged over 5 trials, and were then converted into centimeters, and divided to find the speed of each wheel in cm/s at different duty cycles. The results of the tests are shown in Table 10. Shown in Fig. 20a and b are the velocities plotted against duty cycle for the right and left motor, respectively.

**Table 10:** Speed test results.

Duty Cycle	Velocity (cm/s), Right Wheel	Velocity (cm/s), Left Wheel
0 %	0.00	0.00
10 %	5.37	5.35
20 %	12.89	12.76
30 %	20.25	19.75
40 %	28.01	27.22
50 %	35.76	34.75
60 %	43.31	42.40
70 %	51.25	50.31
80 %	58.77	57.00
90 %	66.41	65.00
100 %	74.02	72.31

Using the obtained polynomial trendlines from the two graphs, the duty cycles to be set for the right and the left motor to move the robot forward and backward at 15 cm/s was found to be 22.28 % and 22.76 %, respectively. These duty cycles were then set to move the robot at the designed speed. The mobile manipulator was then assembled, and further testing was carried out to evaluate its task completion abilities. The results will be discussed in a later section.

### 7.5 Gripping and lifting test

To validate the use of the system in assisting people with motor disabilities, the designed mobile manipulator system is expected to carry various common household items, including a 500 mL water bottle, a Rubik's cube, a medicine box, a TV remote control, and a tennis ball. Prior to testing and evaluating the mobile manipulator system’s lifting and gripping capability, the maximum allowable arm extension distance from the center plate to the tip of the gripper was recorded for each object. It is important to note that the actual arm of the mobile manipulator system can extend further than the value of the maximum allowable arm extension distance. However, exceeding the value of said parameter would prevent the vertical translation while holding the specific items due to bending of the horizontal component. Once the extension distance is set to a value lower than the maximum allowable extension distance, vertical movement for the mobile manipulator system can be achieved. The measured values for the maximum allowable arm extension distance for each object is shown in Table 11.

**Table 11:** Maximum allowable arm extension distance.

Household Item	Maximum Allowable Arm Extension Distance
Water Bottle (500 mL)	42.5 cm
TV Remote Control	53.5 cm
Rubik’s Cube	56 cm
Tennis Ball	57 cm
Medicine Box	59.5 cm
No Load	59.5 cm

After determining the maximum allowable arm extension distance, the mobile manipulator system is then used to hold each of the common household items and vertically move up and down for a total of 10 s in order to assess its ability to grasp and lift objects. This test is repeated 5 times for each item and the number of successful runs is recorded.

Table 12 shows the results of the gripping and lifting capability test. From the table, it was observed that the manipulator system has a 100 % success rate when it comes to gripping and lifting each item. This indicates that the system is effective in handling common household items. It is important to note that out of all the items tested, the TV remote control was held by the manipulator system in an unstable manner. This can be attributed to the TV remote control’s irregular shape that caused some slippage to occur.

**Table 12:** Results of gripping and lifting capability test.

Household Item	Number of Trials	Successful Trials	Success Percentage
Water Bottle (500 mL)	5	5	100 %
Rubik’s Cube	5	5	100 %
Tennis Ball	5	5	100 %
Medicine Box	5	5	100 %
TV Remote Control	5	5	100 %

### 7.6 Task completion test

To validate the effectiveness of the mobile manipulator system to retrieve objects in a home environment, the robotic system was subjected to testing, where the task in the experiment is defined to be the retrieval of an object placed on top of a table at the end of a path in an environment with flat terrain.

The task completion tests were conducted by Users 1 through 7, who utilized gaze-based control to operate the mobile manipulator system. In the initial phase of the study, the focus was placed on evaluating the system's effectiveness and usability in alignment with its intended purpose. It is important to note that none of the participants had disabilities, and the eye-tracking data for Users 5 to 7 were not incorporated into the CNN model. Future phases of the study could be expanded to include participants with motor disabilities, enabling a more comprehensive assessment of the system's user experience and accessibility.

**Fig. 21:**
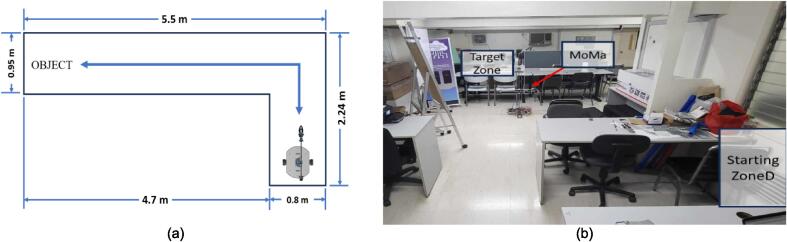
(a) The floor plan of the testing environment. (b) The actual testing environment.

The experimental space consisted of a room with a leveled, flat, tiled floor, furnished with standard tables and chairs. The furniture was arranged to simulate a typical household environment, with ample space between pieces to allow for movement, while still reflecting the complexities of indoor navigation. The robot was remotely operated to navigate this environment from a starting point to a designated location where an object was placed for retrieval, followed by a return to the starting position to safely deposit the object onto a platform. The path included a turn to better emulate real-world indoor environments. A top-view representation of the path and its dimensions is provided in Fig. 21a, while the actual path used in the testing environment is depicted in Fig. 21b. The results of the experiment are summarized in Table 13.

**Table 13:** Results of the object retrieval test for the other objects.

Parameter	Tennis ball	Medicine box	Remote control	Water bottle
Outcome	Success	Success	Failure	Success
Completion time	9 min and 18 s	5 min and 57 s	−	8 min and 27 s
Collision with environment	No occurrence	1 time	No occurrence	No occurrence
Failed grasping attempt	No occurrence	No occurrence	No occurrence	No occurrence
Object dropped along path	No occurrence	No occurrence	No occurrence	No occurrence

Generally, the system is able to successfully complete the mission of picking up various objects from one position and transport it to another position. It was observed that a longer time was needed to complete the task for the tennis ball, as the user took more time to orient the robot so that the gripper would not push the tennis ball and cause it to roll away. On the other hand, the medicine box was completed more quickly as it was smaller and simpler to grasp than the other objects. The remote control was considered a failure, as, due its irregular shape, the gripper was unable to hold it securely, and it slipped off as the robot was moving back along the path. The heaviest object, the water bottle, was successfully retrieved by the robot, with the only challenge being the bottle toppling over when it was released, although it did not fall off the platform.

To reiterate, the system was remotely operated, relying on the user's intuition and feedback to complete the designated tasks. However, challenges arose due to the live video feedback provided by the robotic system, which led to users misjudging the security of the object's placement when deposited. The same limitation is highlighted by the collisions made with the environment, which occurred when the arm met a table at the beginning and end of the path as the robot was made to turn and navigate through a narrower space, as well as the gripper touching the table as a grasping attempt was made. However, it is important to note that collisions were made only 4 times over the 10 trials. Additionally, there were 6 occurrences where the user failed to grasp the object, and either tried to close the gripper when the object was still out of reach, or continually extended the arm when the gripper already encountered the object. Because of the limited view provided by the camera mounted on the robot, there was difficulty in determining how close the object was to the gripper, and whether the object would be properly grasped when the gripper was closed. The addition of sensor systems such as infrared or ultrasonic sensors for obstacle detection and other devices that may help improve the overall interaction with the environment will be recommended for future studies.

Additionally, using a battery with a 5600 mAh capacity, it was observed that testing with continuous operation reduced the battery charge to 50 %. With this, the robotic system can be expected to last 6 h of continuous use. With these results, it has been shown that the robot was capable of carrying out object retrieval tasks. Challenges encountered by the users mostly involved the limitations of the camera. The live video feedback could not provide exhaustive information about the surroundings; thus, the users were unable to gauge how secure the object would be when dropped, and how close the other parts of the manipulator were to obstacles in the surroundings, and how close the object was to the gripper.

### 7.7 Comparison and recommendations

Through the tests detailed previously in this section, the effectiveness and usability of the designed mobile manipulator system, MoMa, were evaluated and validated. In comparison with other existing mobile manipulator systems, MoMa stands out as an accessible and customizable alternative, as it utilizes a webcam-based eye tracking system and off-the-shelf components. It is inexpensive ($632) compared to similar systems such as Dusty [23] (∼$3,000) and Stretch [6] ($17,950 commercially). MoMa can retrieve objects from surfaces as high as 75 cm. The vertical reach of MoMa offers an advantage over systems such as Dusty, which is specifically designed to retrieve objects from the floor; consequently, its construction prevents it from reaching objects above the mobile base. The researchers behind Dusty recommended extending the gripper further and enabling it to rotate towards the ground, while incorporating a compliant gripper to enhance the reliability of object gripping. While the Stretch Mobile Manipulator possesses both capabilities, it is commercially expensive.

MoMa is controlled with the user’s gaze. By using zones for gaze control, the requirements of the eye-tracking system are simplified to a webcam and a gaze prediction training process, without the need for costly systems such as the Gazepoint GP3 ($845) [22]. Its accessibility is also extended to individuals challenged with fine motor movements or upper limb disabilities, as it does not require the use of joysticks or physical controllers as systems like Dusty or Stretch do. As such, the movement of MoMa is fully dependent on the user’s input. Its operation does not involve sensors and software systems necessary for autonomous grasping or navigation. The reliability of the system can be improved by integrating an autonomous grasping sequence as utilized by Dusty, or utilizing visual sensors to gain information on the environment as JACO [5] does. These additional features can be added, although accompanying additional components and costs must be considered.

Results of the validation tests show that the system can perform accurately with an acceptable response time. It is also able to safely bear the design load and grasp household objects without failure or tipping while completing pick and place tasks. However, the results also show some limitations with the robot, namely with its horizontal reach for heavy loads and with feedback regarding the environment. Given that the robotic system is semi-modular, users can opt to include some additional components to alleviate these issues.

The study recommends the following additional components should users see the need to address the aforementioned issues:

● Utilize thicker horizontal supports or increase the number of horizontal bearings. horizontal bearings to four. (Additional cost of 5.3 %)
● Utilize a 360-degree camera, or a wide-lens view for better visual feedback for users
● Utilize infrared or ultrasonic sensors for obstacle detection and avoidance to provide depth perception
● Utilize limit switches for the manipulator to prevent over-extension
● Utilize a different end-effector for specific applications

Further recommendations for future studies could also assess the effectiveness of the implementation of this system by including the participation of individuals with motor disabilities in the validation tests and making improvements through their feedback. Additionally, testing and evaluation of the system in more complex environments that include more types of obstacles is also recommended.

## 8 Ethics statements

All human participants involved in the evaluation of the control system accuracy have given their full signed consent to participate in the study.

## Declaration of generative AI and AI-assisted technologies in the writing process

Generative AI and AI-assisted technologies were not used in the writing process of this manuscript.

## CRediT authorship contribution statement

**James Dominic O. Go:** Writing – review & editing, Writing – original draft, Software, Methodology, Investigation, Conceptualization. **Neal Garnett T. Ong:** Writing – review & editing, Writing – original draft, Investigation, Conceptualization. **Carlo A. Rafanan:** Writing – original draft, Methodology, Investigation, Conceptualization. **Brian G. Tan:** Writing – review & editing, Writing – original draft, Methodology, Investigation, Conceptualization. **Timothy Scott C. Chu:** Writing – review & editing, Supervision, Formal analysis, Conceptualization.

## Declaration of competing interest

The authors declare that they have no known competing financial interests or personal relationships that could have appeared to influence the work reported in this paper.
